# Whole transcriptome landscape in HAPE under the stress of environment at high altitudes: new insights into the mechanisms of hypobaric hypoxia tolerance

**DOI:** 10.3389/fimmu.2024.1444666

**Published:** 2024-09-12

**Authors:** Qiong Li, Fujin Fang, Chuanli Yang, Dong Yu, Qianhui Gong, Xiaobing Shen

**Affiliations:** ^1^ Key Laboratory of Environmental Medicine Engineering, Ministry of Education, School of Public Health, Southeast University, Nanjing, China; ^2^ Department of Epidemiology and Health Statistics, School of Public Health, Southeast University, Nanjing, China

**Keywords:** transcriptome, sequence analysis, circRNA, lncRNA, miRNA, high-altitude

## Abstract

**Background:**

High altitude pulmonary edema (HAPE) is an idiopathic, noncardiogenic form of pulmonary edema that occurs at high altitudes. It is characterized by a severe clinical course and carries a significant mortality risk. Despite its clinical relevance, the molecular mechanisms underlying HAPE are not well understood.

**Methods:**

We conducted whole-transcriptome RNA sequencing on blood samples from 6 pairs of HAPE patients and healthy controls to identify differentially expressed (DE) mRNAs, miRNAs, circRNAs, lncRNAs, along with alternative splicing (AS) events, gene fusions, and novel transcripts. To explore the regulatory dynamics, we constructed ceRNA networks and analyzed immune cell infiltration patterns, further annotating the biological functions of these transcripts. For empirical validation, we selected five circRNAs from the ceRNA network and conducted RT-qPCR on 50 paired samples. Additionally, we assessed the correlations between circRNA expression levels and clinical data to evaluate their diagnostic potential.

**Results:**

We observed 2,023 differentially expressed mRNAs (DEmRNAs), 84 DEmiRNAs, 200 DEcircRNAs, and 3,573 DElncRNAs. A total of 139 ‘A3SS’ events, 103 ‘A5SS’ events, 545 ‘MXE’ events, 14 ‘RI’ events, and 1,482 ‘SE’ events were identified in the AS events analysis between the two groups. Two ceRNA networks were constructed. T cells, follicular helper, and Macrophages M1 cells exhibited the strongest positive correlation (R=0.82), while naive B cells and memory B cells demonstrated the strongest negative correlation (R=-0.62). In total, the expression of three circRNAs was significantly different in a larger cohort. Hsa_circ_0058497, hsa_circ_0081006, and hsa_circ_0083220 demonstrated consistent with the RNA sequencing results. These three circRNAs strongly correlate with clinical indicators and exhibit potential as diagnostic biomarkers. Finally, we verified five genes (CXCR4, HSD17B2, ANGPTL4, TIMP3, N4BP3) that were differentially expressed in endothelial cells under normoxia and hypoxia through bioinformatics and RT-qPCR analyses.

**Conclusion:**

This study elucidates the differential expression of coding and non-coding RNAs (ncRNAs) in HAPE, identifies new transcripts and genes, and enhances our understanding of the transcriptional characteristics of HAPE. Moreover, it highlights the potential role of circRNAs in advancing the diagnosis and treatment of HAPE.

## 1 Introduction

### 1.1 High altitude pulmonary edema is a potentially fatal condition

People living at high altitude have adapted to low oxygen levels over extended periods, developing new adaptive traits to cope with these environmental conditions (1). Residents of the Qinghai-Tibetan Plateau in China, situated approximately 3,000 meters above sea level, have developed unique physiological characteristics to adapt to the extreme high-altitude environment. These adaptations include higher hemoglobin concentrations, increased oxygen saturation of the blood, and an enhanced rate of energy metabolism, enabling them to cope with reduced oxygen levels effectively (2). However, the extreme low pressure and reduced oxygen levels in the Qinghai-Tibet Plateau of China pose significant threats and challenges to tourists and workers from lower altitudes. Rapid ascent without proper acclimatization can lead to serious and potentially fatal conditions for those not accustomed to high-altitude environments. Mortality from untreated HAPE is around 50% (3). However, HAPE is reversible if the patient receives adequate treatment in a timely manner, such as acetazolamide (4), salmeterol (5), nifedipine (6), and dexamethasone (7). These treatments have proven effective in reducing relapse and progression, yet they are sometimes avoided due to potential side effects. Despite years of extensive research, treatment options for HAPE remain limited and challenging. Consequently, there is a critical need to identify novel mechanisms and therapeutic targets to improve management strategies for this condition.

### 1.2 Whole transcriptome sequencing studies on HAPE are important but relatively rare

While several studies have analyzed transcriptomic profiles in high-altitude lung injuries, few have specifically characterized changes in non-coding RNAs (ncRNAs) during the onset of HAPE. Beyond their potential as diagnostic biomarkers, ncRNAs may also serve as targets or tools in novel therapeutic strategies. MicroRNAs (miRNAs) in particular play a crucial role in gene regulation, effectively silencing gene expression through translational repression or mRNA degradation by binding to the 3’-untranslated regions of their target mRNAs (8). Accumulating evidence suggests a role for peripheral blood hypoxia-related miRNAs in the pathogenesis of high-altitude hypoxic lung injury. For example, miR-124-3p is implicated in the pathophysiology of HAPE by inhibiting the expression of key genes (9). MiR-203a-3p restricts the angiogenic capacity of pulmonary microvascular endothelial cells *in vitro*, directly suppressing VEGF-A translation and indirectly reducing Notch1, VEGFR2, and Hes1 levels (10).

Long non-coding RNAs (lncRNAs), a newly recognized class of non-coding transcripts longer than 200 nucleotides, are involved in the development and progression of HAPE because they can regulate gene expression at the transcriptional, post-transcriptional, and epigenetic levels (11). A study found that MALAT1, one of the lncRNAs, controls endothelial cell phenotype transition and regulates vascular growth in a rat model, significantly increasing under hypoxia (12). However, few studies on lncRNAs associated with HAPE are available in the published literature.

Circular RNA (circRNA) represents a newly identified class of non-coding RNA, characterized by backsplicing of multiple exons (13). Its high expression levels in various tissues render it a superior biomarker candidate compared to linear RNAs. Considering the involvement of circRNAs in HAPE progression, we aimed to determine differential expression of selected circRNAs and assess their potential use as novel blood-based biomarkers for disease evaluation. Although some reports suggest that circRNAs are implicated in various diseases, their involvement in HAPE has not yet been documented. Similarly, research on alternative splicing (AS) events, gene fusion analysis, and novel transcript prediction remains sparse in the field of high-altitude research. To assess how extensively the transcriptome is perturbed in HAPE and to address the gaps in HAPE research, we conducted a comprehensive transcriptome analysis via RNA sequencing. This approach also facilitates the detection of RNA editing events characterized by disease-specific features. The integrative analysis of whole transcriptome sequencing, the construction of a ceRNA (circRNA/lncRNA-miRNA-mRNA) interaction axis, and clinical correlation analysis of circRNA represent significant strengths of our study. Additionally, ceRNAs possessing similar miRNA response elements (MREs) can bind miRNAs in a regulatory fashion akin to that of miRNA regulation (14). Predicting AS events, gene fusions, and novel transcripts in this study significantly enhances the comprehensive understanding of HAPE pathogenesis.

### 1.3 HAPE has been linked to immune cell infiltration and hypoxia-induced dysfunction of vascular endothelial cells

Among the various selective pressures at high altitudes, hypobaric hypoxia presents the most significant challenge, shaping the physiological acclimatization of low-altitude dwellers. HAPE is often accompanied by an inflammatory response, with hypobaric hypoxia considered the primary cause (15). Meanwhile, acute hypobaric hypoxia induces pulmonary vasoconstriction, while prolonged exposure results in sustained pulmonary vasoconstriction and vascular remodeling, culminating in HAPE (16). Thus, we employed several bioinformatics approaches, including immune cell infiltration and analysis of endothelial cell GEO datasets, to further explore the pathogenesis of HAPE in vascular endothelial cells.

The aim of this study is to identify differentially expressed transcriptional features between patients with HAPE and controls through whole transcriptome sequencing. Additionally, the interaction between genetic and environmental factors is hypothesized to be a primary cause of HAPE (17) (18).We hypothesize that epigenetic indicators, specifically circRNAs, lncRNAs, and miRNAs, can reflect the unique characteristics of patients with high altitude pulmonary edema (HAPE). This study focuses on circRNAs, validating their potential as disease diagnostic biomarkers and exploring their viability as novel therapeutic targets for HAPE. It aims to uncover the clinical value and molecular mechanisms of circRNAs in the disease context. Additionally, this study integrates RNA sequencing data with the GEO dataset to investigate potential mechanisms of immune cell infiltration and endothelial cell dysfunction in the pathophysiology of HAPE.

## 2 Materials and methods

### 2.1 Study design

**Figure 1:**
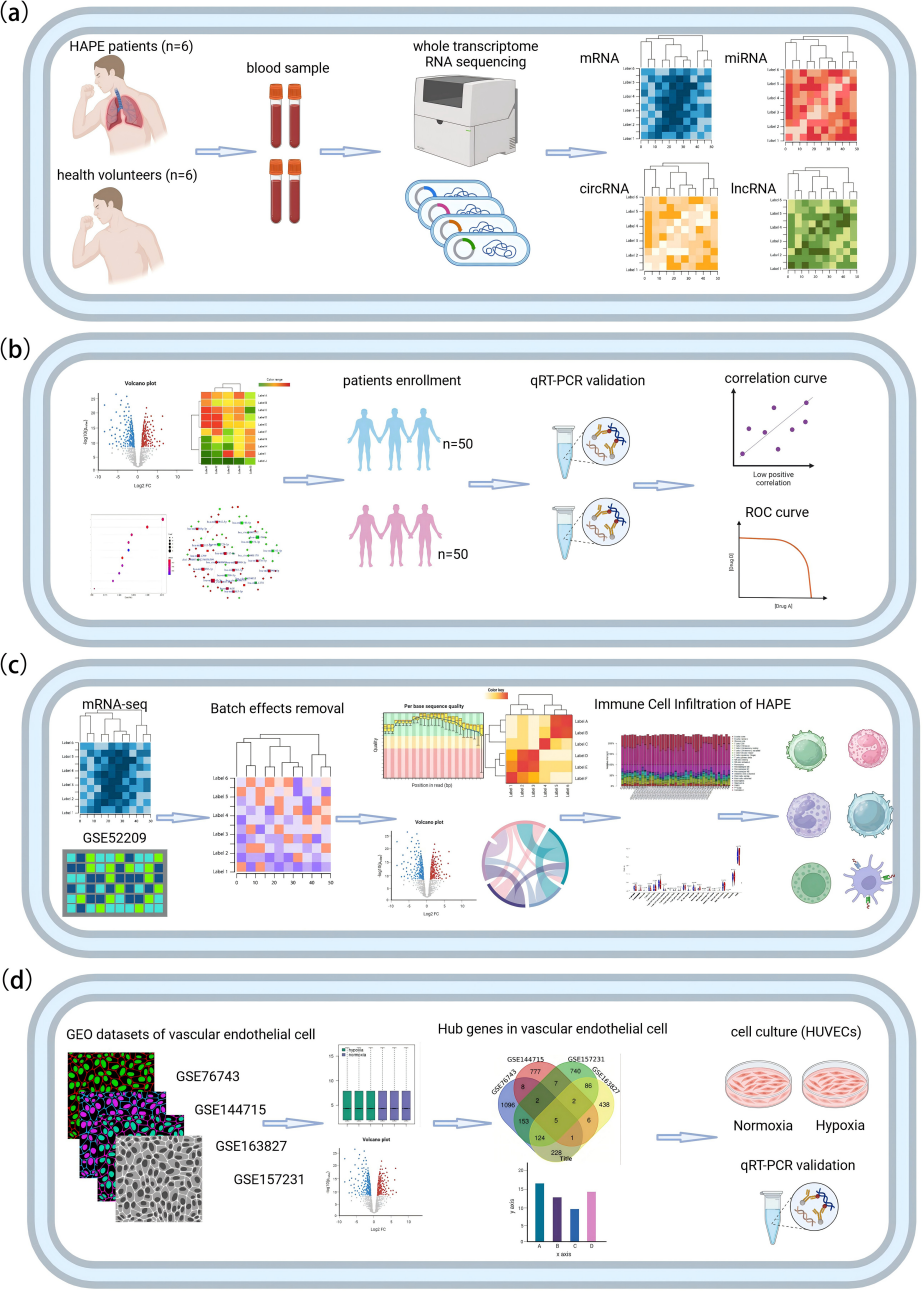
The flowchart of this study. **(A)** RNA high-throughput sequencing. **(B)** Bioinformatics analysis and validation of circRNA in independent samples. **(C)** Analysis of immune cell infiltration in HAPE. **(D)** Identification and validation of HAPE-related genes in hypoxic endothelial cells.

1. The first part of the study is a case-control analysis, with patients suffering from high altitude pulmonary edema (HAPE) constituting the case group, and healthy individuals serving as the control group. We employed RNA high-throughput sequencing to identify differentially expressed candidate mRNAs, miRNAs, circRNAs, lncRNAs, as well as alternative splicing (AS) events, fusion genes, and novel transcripts in the discovery set, which included 6 HAPE patients and 6 healthy controls (**Figure 1A**).
2. In the second part of the study, we constructed gene co-expression and ceRNA networks. We then validated the differential expression of candidate circRNAs using RT-qPCR in a separate validation set comprising 50 HAPE patients and 50 healthy controls. To evaluate the diagnostic value of these circRNAs for HAPE, we employed ROC curve analysis. Additionally, we conducted correlation analysis between these circRNAs and the clinical phenotype of HAPE patients to assess the potential clinical value of these circRNAs in diagnosing and understanding HAPE (**Figure 1B**).
3. The third part of the study involves integrating the results from mRNA sequencing with the HAPE dataset from the GEO database to investigate the role of immune cell infiltration in the onset and progression of HAPE (**Figure 1C**).
4. The fourth part of the study focuses on mining hypoxia-related endothelial cell datasets from the GEO database. We combined and analyzed these datasets with our sequencing data to identify key genes associated with endothelial cell dysfunction. Subsequent RT-qPCR experiments were conducted using a hypoxic human umbilical vein endothelial cell (HUVEC) model to verify the expression of these key genes (**Figure 1D**). The research process is shown in **Figure 1**.

### 2.2 Participants

Patients were recruited from Houbei Hospital in Lhasa, Tibet, between February 2021 and July 2023. Additional donor controls were sourced from healthy volunteers who traveled to Tibet. All participants and health volunteers were Han Chinese, aged 18 to 65 years.

The inclusion criteria include (1) Han population, requiring Han people who have lived in the plains for more than three generations and have entered the plateau for the first time; (2) People aged 18 and above; (3) HAPE patients: a population clinically diagnosed with high altitude pulmonary edema. (4) Healthy donors: Healthy individuals who do not suffer from serious illnesses. (5) Women who are not pregnant or breastfeeding. (6) People who have not taken any medication before entering the plateau.

Exclusion criteria included (1) long-term residence in high-altitude areas, (2) serious physical illnesses such as neurogenic, endocrine, metabolic, autoimmune, cardiovascular, and cerebrovascular diseases, (3) non-Han Chinese ancestry within three generations, and (4) unsuitability or inability to complete the study for other reasons. The study was approved by the XIZANG Center for Disease Control and Prevention Ethics Review Committee (reference number: 2023-001), and conforms to the Declaration of Helsinki. All participants provided written informed consent.

### 2.3 HAPE diagnosis and sample collection

HAPE diagnosis was conducted by specialty-trained physicians and radiologists based on clinical information, including breathlessness, bluish skin, dry cough on exercise, pink foamy sputum, difficulty breathing when lying down, and fever, as well as chest X-ray findings. On the first day of hospitalization, prior to initiating any treatment measures, HAPE patients provided 5 mL of anticoagulated blood samples. Similarly, healthy volunteers collected a single 5 mL blood sample on their third day in high-altitude areas. Each sample was immediately placed in a PAXgene RNA Tube, specifically designed for whole transcriptome sequencing. Following collection, all samples were stored in an -80°C freezer for future analysis. Concurrently with sample collection, the study also gathered comprehensive clinical data from the participants. This included the frequency of their visits to the plateau, past medical history, family medical history, as well as their height, weight, and routine blood and biochemical indicators. To ensure scientific reliability and minimize data entry errors, a dual-person, dual-entry method was employed for recording this information.

### 2.4 RNA high-throughput sequencing

According to the manufacturer’s instructions, total RNA was extracted using the PAXgene Blood RNA Kit (Qiagen) and QIAcube (Qiagen) automated processor under standardized conditions. Genesky Biotechnology Inc. (Shanghai, China) generated a sequencing library using standard Illumina protocol and performed high-throughput RNA sequencing. Please refer to the **Supplementary Materials** for details. All sequencing data have been deposited in the GEO database (https://www.ncbi.nlm.nih.gov/geo/) under accession GSE260910.

### 2.5 Small RNA sequencing

This study delineated the expression profiles of mRNAs, miRNAs, circRNAs, and lncRNAs from the same samples and performed subsequent bioinformatics analyses. Please refer to the **Supplementary Materials** for details.

### 2.6 Bioinformatics analysis of RNA sequencing data

To delve into the interaction mechanisms among differentially expressed circRNAs, lncRNAs, miRNAs, and mRNAs, we constructed gene co-expression networks as well as circRNA-miRNA-mRNA and lncRNA-miRNA-mRNA interaction networks. These networks were designed to explore the competing endogenous RNA (ceRNA) hypothesis, which posits that non-coding RNAs can regulate each other through shared miRNA response elements. Additionally, we conducted Gene Ontology (GO) and Kyoto Encyclopedia of Genes and Genomes (KEGG) functional enrichment analyses to better understand the biological implications of these interactions. Details of these processes are provided in the **Supplementary Materials**.

### 2.7 Sample size estimation

We calculated the power of 6 pairs of samples and the sample size for subsequent independent sample validation using online websites. Please refer to the **Supplementary Materials** for the calculation process.

### 2.8 GEO dataset collection

The mRNA expression profiles were collected from the GEO database. In GSE52209, 17 HAPE patients and 14 controls were selected to explore immune infiltration in combination with our transcriptome sequencing data. To further analyze the key differentially expressed genes in endothelial cells under hypoxia and normoxia, we downloaded GSE76743, GSE144715, GSE157231, and GSE163827. To retrieve DEGs, volcano plots, and heatmap plots between the four datasets, we used the differential gene expression analyses on an online analysis tool, GEO2R, from the GEO database. **Supplementary Table S1** lists all other GEO datasets utilized for the bioinformatics analyses in the article.

### 2.9 Immune cell infiltration landscapes

The R packages ‘ComBat’ and ‘sva’ were used to remove batch effect as samples were clustered into two groups (HAPE and controls) corresponding to the two sequencing batches. Potential batch effects were visualized by the construction of a PCA plot by batch and disease type with the use of the R package ‘limma’. For the analysis of immune cell proportions in blood samples from HAPE patients, immune cell infiltration analyses were explored with CIBERSORT algorithms, with the following packages in the R software: ‘e1071’, ‘parallel’ and ‘preprocessCore’.

### 2.10 Cell culture

VECs are important cells closely linked to how HAPE develops and progresses. Therefore, human umbilical vein endothelial cells (HUVECs) were selected for cultivation under normoxia (CO^2^ 5%, O^2^ 21%, 24h) and hypoxic (CO^2^ 5%, O^2^ 1%, 24h) conditions in this study. Hypoxia exposure simulation was performed in a hypobaric hypoxic chamber (Hangzhou Aipo Instrument Co., Ltd, China). HUVEC were cultured in DMEM, 10% fetal calf serum (FCS), and 1% Glutamax (Gibco).

### 2.11 Quantitative real-time reverse transcription PCR

Total RNA was extracted using Trizol (Invitrogen). StarScript II First-strand cDNA Synthesis Kit-II (GenStar, Beijing, China) was used to reverse transcribe the RNA into complementary DNA (cDNA). The qRT-PCR was performed using 2 × RealStar Green Fast Mixture with ROX (GenStar, Beijing, China) on a 384-well plate qRT-PCR device (ABI 7500; Applied Biosystems, USA). The 2^-ΔΔCT^ method with GAPDH as an internal reference was used to determine gene expression levels. The results are presented as the mean ± SE and were graphed using GraphPad Prism software (version 10.0). Please refer to the **Supplementary Materials** for the experimental conditions of rt-qPCR. The primers were synthesized by the Beijing Genomics Institute (BGI, Beijing, China), as described in **Supplementary Table S2**.

### 2.12 Statistical analysis

The statistical analysis of the demographic data was carried out using the R software (4.2.1). The chi-squared test was used for categorical variables. Continuous variables were analyzed using independent samples t-test or Mann-Whitney U test. The correlations between variables were analyzed with the Pearson correlation tests. A *P* value of less than 0.05 was considered statistically significant.

## 3 Results

### 3.1 Differential expression analysis of mRNA, miRNA, circRNA, and lncRNA in HAPE

**Figure 2:**
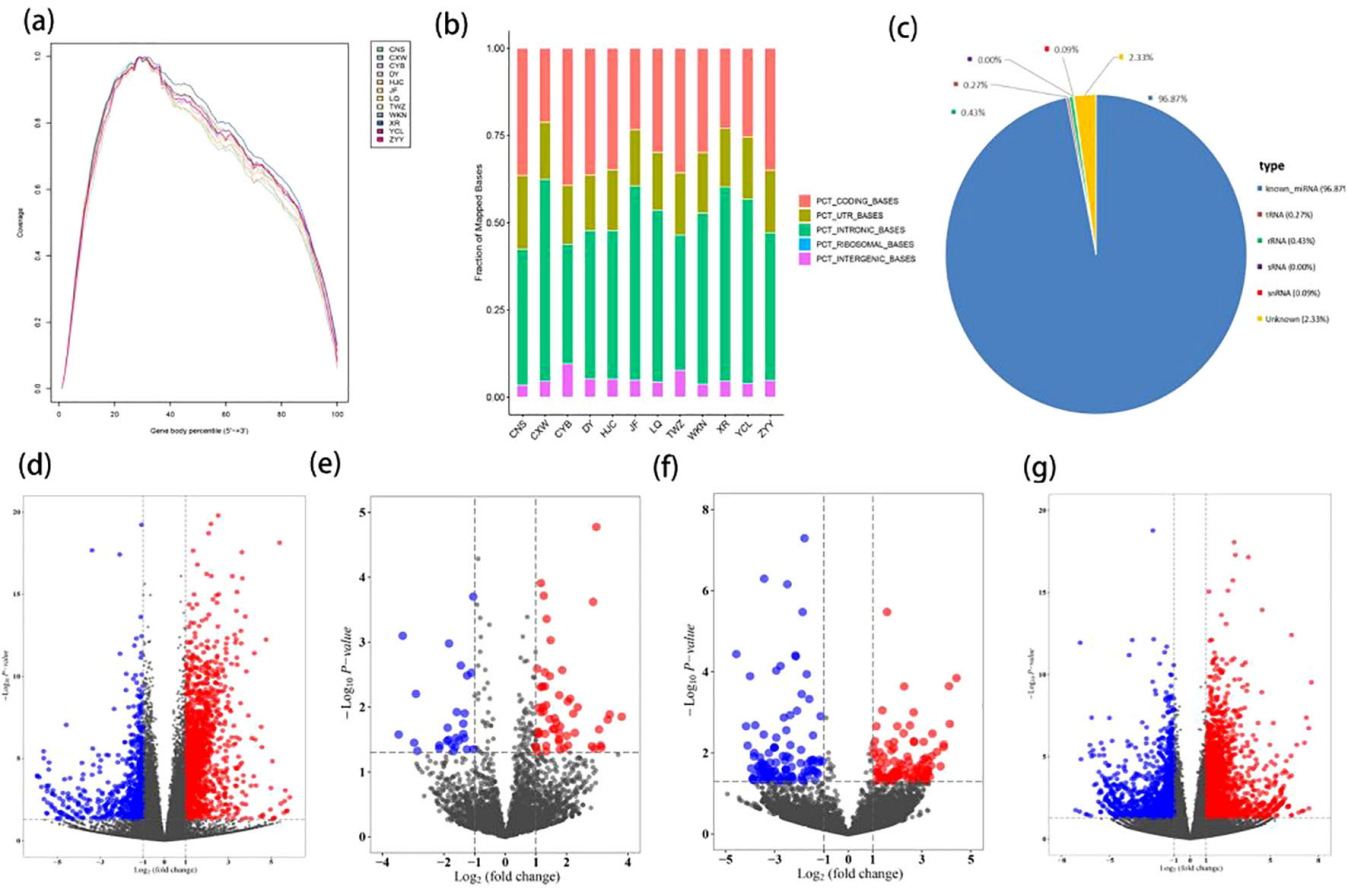
Differential expression analysis of mRNA, miRNA, circRNA, and lncRNA in HAPE. **(A)** A analysis of genomic coverage in 12 samples. **(B)** Distribution bar chart of all sample Reads in different regions of the reference genome. PCT_ CODING_ BASES: The proportion of bases aligned to the coding region; PCT_ UTR_ BASES: The proportion of bases aligned to the UTR region; PCT_ INTRONIC_ BASES: The proportion of bases aligned to introns; PCT_ INTERGENIC_ BASES: The proportion of bases aligned to the intergenic region. **(C)** Pie chart of small RNA classification annotation. **(D–G)** The volcano plots of the DEmRNA, DEmiRNA, DEcircRNA, DElncRNA. **(D)** DEmRNA; **(E)** DEmiRNA; **(F)** DEcircRNA; **(G)** DElncmRNA.

**Table 1** lists the baseline characteristics of the study subjects, and it can be observed that the gender, age, height, weight, and BMI of the two groups are matched. The sequence coverage of all genes in the 5’ to 3’ regions of the sample indicated homogeneity in the sequencing experiment results (**Figure 2A**). Filtered reads were aligned to a reference database using STAR software, demonstrating high sequencing quality, excellent selection of reference genomes, and reliable subsequent analysis quality (**Figure 2B**). The number and proportion of sequences annotated to various small RNA types (such as known miRNA, tRNA, rRNA, snoRNA, snRNA) genome positions in the trimmed sequences comparison with known mature miRNA and non-coding RNA are detailed in **Figure 2C**, demonstrating the high quality of sequencing data. In comparison with the control group, we identified 2023 differentially expressed mRNAs (1386 upregulated and 637 downregulated), 84 differentially expressed miRNAs (55 upregulated and 29 downregulated), 200 differentially expressed circRNAs (97 upregulated and 103 downregulated), and 3573 differentially expressed lncRNAs (1957 upregulated and 1616 downregulated) in the HAPE groups. These findings are visualized in volcano plots (**Figures 2D–G**) and heatmaps (**Supplementary Figures S1A–D**). The top 10 differentially expressed mRNAs, miRNAs, circRNAs, and lncRNAs are listed in **Supplementary Tables S3**-**S6**.

**Table 1:** Baseline characteristics of sequencing samples.

	HAPE Patients	Controls	P
age(years)	30.67 ± 5.68	29.83 ± 3.601	0.7677
sex ration(males:females)	1:1	1:1	
Height(cm)	165.2 ± 9.432	164.3 ± 7.521	0.869
Weight(kg2)	58.75 ± 8.777	55.63 ± 8.102	0.537
BMI(Kg/cm2)	21.41 ± 1.276	20.57 ± 2.401	0.468

### 3.2 Functional enrichment analysis of GO and KEGG

To investigate HAPE-related pathways among differentially expressed mRNAs, miRNAs, circRNAs, and lncRNAs, we conducted enrichment analyses using the KEGG pathway and the GO biological process annotations through Fisher’s exact test. The analysis of DEmRNAs revealed significant enrichment in molecular functions (MF) such as haptoglobin binding, GABA receptor binding, and extracellular matrix binding; cellular components (CC) such as hemoglobin complex, tertiary granule lumen, and specific granule lumen; and biological processes (BP) such as protoporphyrinogen IX metabolic process, porphyrin-containing compound biosynthetic process, and tetrapyrrole biosynthetic process (**Supplementary Figure S2A**).

To further elucidate the differential RNA transcriptome associated with biological processes between HAPE and the control group, KEGG pathway analyses were performed. The results indicated that the most significantly altered pathways among DEmRNAs included Mitophagy, Glycine, Serine and Threonine Metabolism, Ferroptosis, Ubiquitin-Mediated Proteolysis, Porphyrin Metabolism, Nitrogen Metabolism, and Cytokine-Cytokine Receptor Interaction, among others (**Supplementary Figure S2E**). Additional results are detailed in **Supplementary Figures S2B–D**, **Supplementary Figures S2F–H**, and the **Supplementary Materials**.

### 3.3 AS events analysis, gene fusions analysis, and prediction of novel transcripts

A total of 139 ‘A3SS’ events, 103 ‘A5SS’ events, 545 ‘MXE’ events, 14 ‘RI’ events, and 1482 ‘SE’ events in alternative splicing (AS) analysis between the two groups were identified. The most significant differences in AS events can be seen in **Supplementary Table S7**. Because fused genes often exhibit similar functions, the identification of fusion events can aid in inferring gene functions (19). Consequently, a total of 8 pairs of fusion genes were identified through screening in the HAPE group samples, including HBA2-MT-RNR2, FBXO30-EPM2A, and RNF138-RNF125 (**Supplementary Table S8**). Moreover, the human transcriptome still harbors a large number of undiscovered new transcripts. In this study, we identified 219 novel transcripts that were specifically expressed in humans. The top 10 novel transcripts are listed in **Supplementary Table S9**. The annotation of novel transcripts significantly increased the number of known transcripts expressed in the HAPE groups.

### 3.4 Co-expression gene and ceRNA network

**Figure 3:**
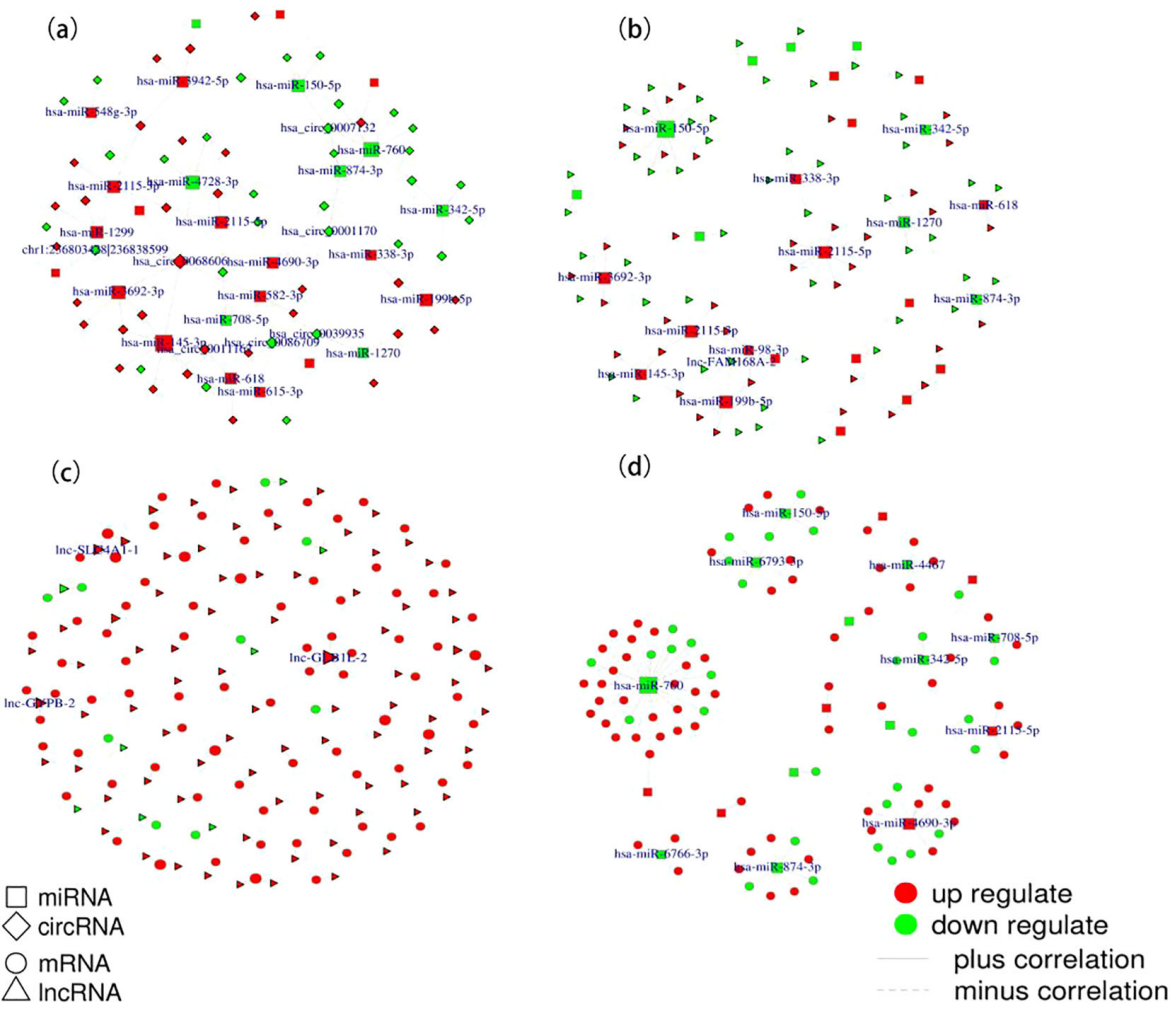
TOP 100 Co-expression networks. **(A)** circRNA-miRNA co-expression network. **(B)** lncRNA-miRNA co-expression network; **(C)** lncRNA-mRNA_Cis co-expression network; **(D)** miRNA-mRNA co-expression network.

Co-expression networks are instrumental in linking genes of unknown function to biological processes, prioritizing candidate disease genes, and identifying transcriptional regulatory programs. **Figure 3** displays the top 100 co-expressed gene networks. Utilizing criteria of Pearson correlation coefficients (R) above 0.8 and P values below 0.05, the circRNA-miRNA co-expression network includes 36 pairs (**Figure 3A**), the lncRNA-miRNA co-expression network comprises 1729 pairs (**Figure 3B**), the lncRNA-mRNA-Cis co-expression network encompasses 188 pairs (**Figure 3C**), and the miRNA-mRNA co-expression network involves 16 pairs (**Figure 3D**). Detailed results are provided in the **Supplementary Tables S10**-**S13**.

**Figure 4:**
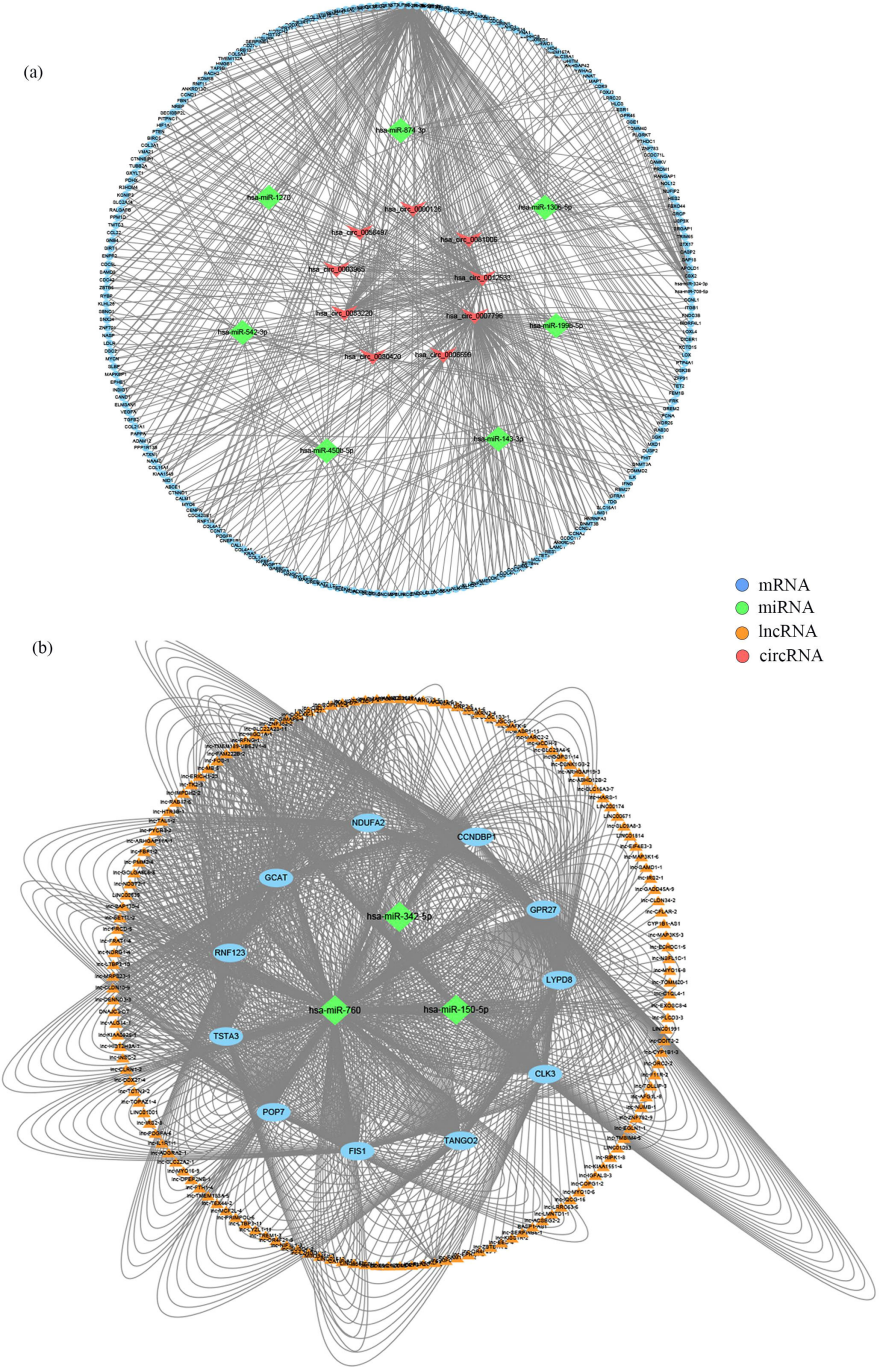
Construction of ceRNA networks. **(A)** A circRNA-miRNA-mRNA ceRNA network. **(B)** A lncRNA-miRNA-mRNA ceRNA network.

Based on the ceRNA hypothesis, we constructed circRNA-miRNA-mRNA and lncRNA-miRNA-mRNA regulatory networks to reveal the roles and interactions of mRNAs, miRNAs, circRNAs, and lncRNAs (**Figures 4A, B**). The circRNA-related ceRNA networks indicated that the top five ceRNAs may bind to three miRNAs and regulate the expression of three mRNAs (**Table 2**). Within the circRNA-related ceRNA networks, PAPPA, ARHGAP42, and SRGAP1 have particularly attracted our attention. The lncRNA-related ceRNA network showed that the top five ceRNAs may bind to three miRNAs and regulate the expression of eleven mRNAs (**Table 3**).

**Table 2:** CircRNA-ceRNA network.

circRNA	circRNA_regulation	miRNA	miRNA_regulation	mRNA	mRNA_regulation
hsa_circ_0012533	Down	hsa-miR-143-3p	Up	PAPPA	Down
hsa_circ_0006699	Down	hsa-miR-143-3p	Up	PAPPA	Down
hsa_circ_0081006	Up	hsa-miR-708-5p	Down	ARHGAP42	Up
hsa_circ_0058497	Up	hsa-miR-708-5p	Down	ARHGAP42	Up
hsa_circ_0083220	Up	hsa-miR-1306-5p	Down	SRGAP1	Up

**Table 3:** LncRNA-ceRNA network.

lncRNA	lncRNA_regulation	miRNA	miRNA_regulation	mRNA	mRNA_regulation
lnc-SLC22A23-11	Up	hsa-miR-760	Down	CLK3、TANGO2、FIS1、POP7、TSTA3、RNF123、GCAT、NDUFA2、CCNDBP1	Up
LINC01991	Up	hsa-miR-150-5p	Down	GPR27	Up
lnc-TAL1-2	Up	hsa-miR-760	Down	CLK3、TANGO2、FIS1、POP7、TSTA3、RNF123、GCAT、NDUFA2、CCNDBP1	Up
lnc-LRRC32-5	Up	hsa-miR-342-5p	Down	LYPD8	Up
lnc-GCDH-3	Up	hsa-miR-342-5p	Down	LYPD8	Up

### 3.5 Immune cell infiltration analysis

**Figure 5:**
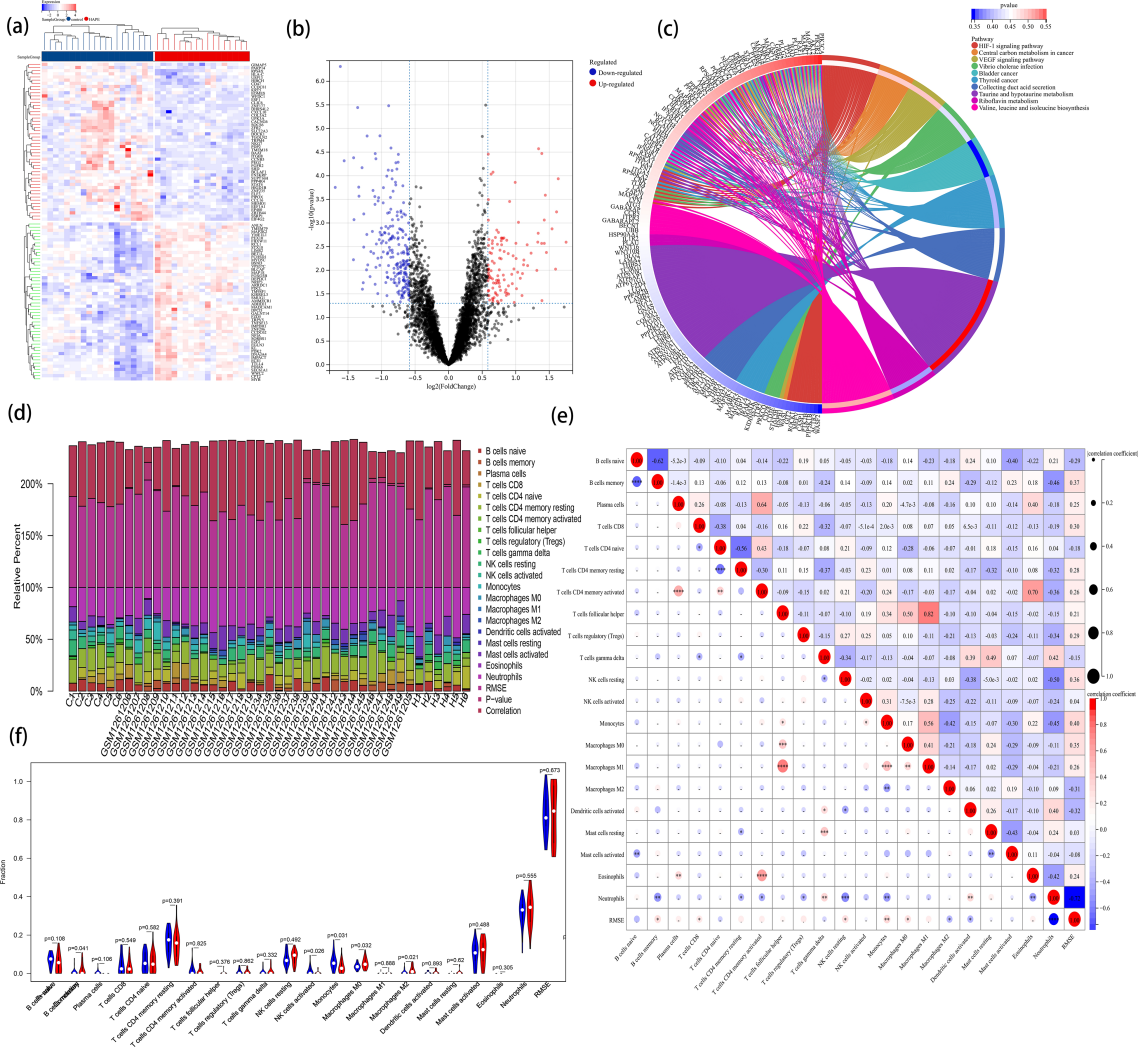
The landscape of immune infiltration in HAPE. **(A)** the heatmap of DEGs within the entire data set; **(B)** the volcano plot of DEGs within the entire data set; **(C)** KEGG pathway enrichment analysis; **(D)** A column chart of the percentage of immune cells at each sample; **(E)** Correlation heatmap depicting correlations between infiltrated immune cells in HAPE; **(F)** Violin plot visualizing the differentially infiltrated immune cells.

To augment the sample size for this immune cell infiltration analysis, sequence data of HAPE from GSE52209 were incorporated, sourced from the GEO database. The data were pre-processed and normalized to mitigate batch effects (**Supplementary Figure S3**). Volcano plots and heatmaps depicted the differentially expressed profiles of DEGs across the entire dataset (**Figures 5A, B**). KEGG enrichment analysis identified the HIF-1 and VEGF signaling pathways as predominantly enriched in pathways associated with HAPE (**Supplementary Table S14**, **Figure 5C**).

To elucidate the immune mechanisms in the HAPE group and controls, immune cell infiltration analyses were conducted using CIBERSORT. The percentages of 22 types of immune cells in each sample were quantified in a column chart as indicated in **Figure 5D**. The proportions of immune cells varied significantly between groups. Examination of potential relationships among the 22 immune cell types revealed a weak to moderate correlation among the ratios of different immune cell subpopulations infiltrating HAPE (**Figure 5E**). T cells follicular helper (Tfh) and Macrophages M1 exhibited the strongest positive correlation (R = 0.82), while naive B cells and memory B cells exhibited the strongest negative correlation (R = 0.62). Additionally, activated T cells CD4 memory demonstrated a strong positive correlation with Eosinophils (R = 0.70). Compared with the control group, the HAPE group had higher levels of memory B cells, Macrophages M0, and Macrophages M2 (P<0.05). However, activated NK cells and Monocytes were significantly lower in the HAPE group (P<0.05) (**Figure 5F**).

### 3.6 Differentially expressed circRNAs of the ceRNA network in the independent validation set

**Figure 6:**
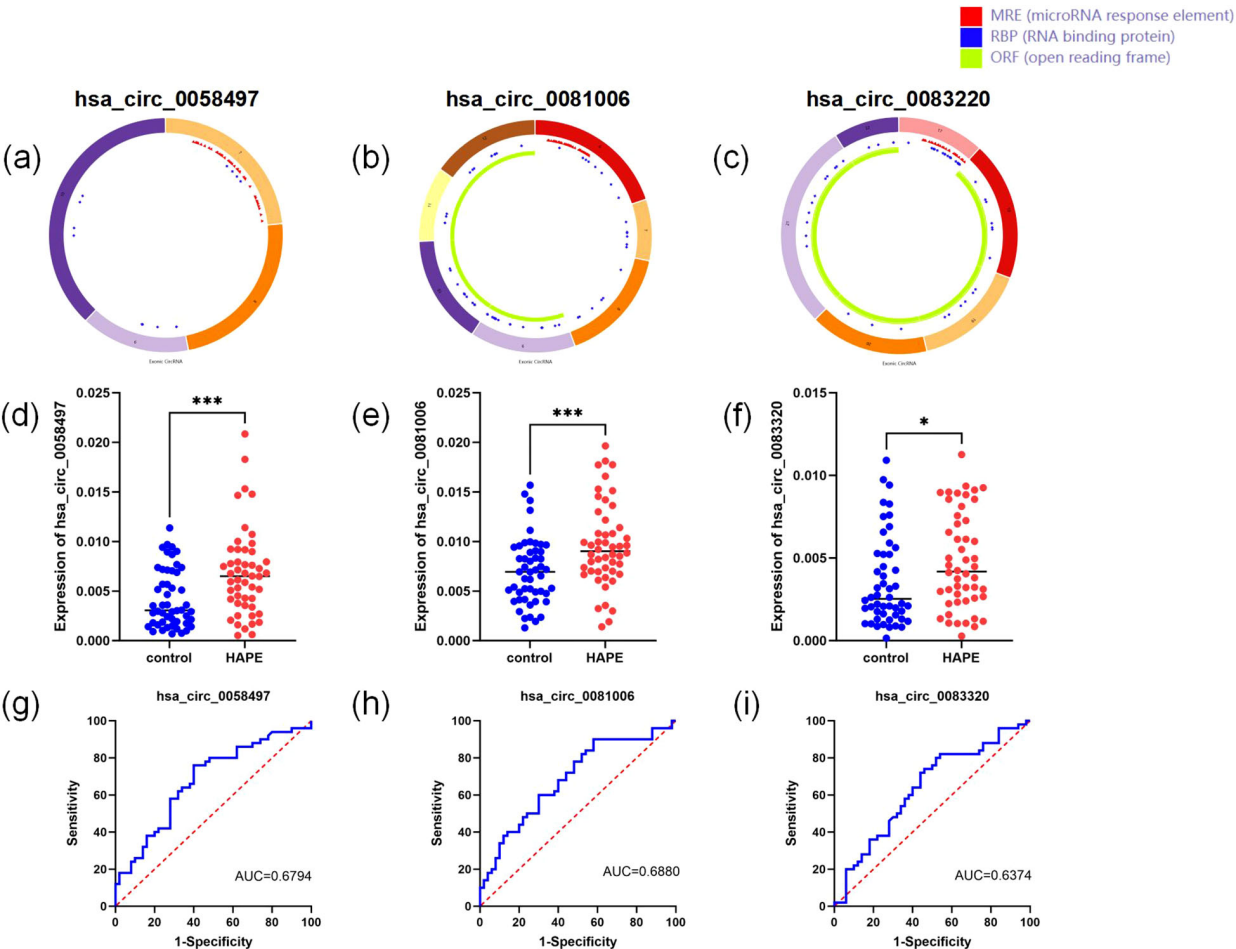
Validation of differentially expressed circRNAs in the ceRNA network. **(A–C)** the structural loop graphs of 3 circRNAs were obtained through the CSCD database (http://gb.whu.edu.cn/CSCD/). **(D–F)** Expression of three circRNAs based on rt-qPCR. **(G–I)** ROC curves of three circRNAs. * P <0.05, ** P<0.01, ***P<0.001.

Given that circRNAs are involved in critical cellular processes, our research team became interested in how circRNAs affect the process of HAPE. Consequently, we performed rt-PCR on 50 pairs of HAPE and control samples, targeting five differentially expressed circRNAs identified in the ceRNA network. For circRNA expression analysis, linear RNAs were eliminated using RNase R treatment, and circRNA-specific primers were devised for subsequent experiments. Although primer design was unsuccessful for hsa_circ_0012533 and hsa_circ_0006699, primers for the other three circRNAs were successfully developed and utilized in rt-qPCR experiments (**Figures 6A–C**). Notably, all three circRNAs showed statistically significant differences between the two groups (**Figures 6D–F**). Additionally, receiver operating characteristic (ROC) curves were constructed to assess the diagnostic potential of these circRNAs. The AUC values for hsa_circ_0058497, hsa_circ_0081006, and hsa_circ_0083220 were 0.6794 (95% CI: 0.5741-0.7847), 0.6880 (95% CI: 0.5839-0.7921), and 0.6374 (95% CI: 0.5280-0.7468), respectively (**Figures 6G–I**). These results suggest that these circRNAs may possess significant predictive value. Subsequently, we analyzed the correlations between the levels of these three circRNas and their association with clinical variables. As illustrated in **Table 4**, the expression levels of hsa_circ_00058497 were negatively correlated with hemoglobin (HGB, r = -0.679, P < 0.01), red blood cells (RBC, r = -0.601, P < 0.01), hematocrit (HCT, r = -0.547, P < 0.01), and indirect bilirubin (IBIL, r = -0.334, P < 0.01). The level of hsa_circ_0081006 was significantly positively correlated with white blood cells (WBC, r = 0.477, P < 0.01). Additionally, hsa_circ_0083220 was negatively associated with hemoglobin (HGB, r = -0.412, P < 0.01), red blood cells (RBC, r = -0.440, P < 0.01), and hematocrit (HCT, r = -0.361, P < 0.01). Here, only results with a correlation coefficient greater than 0.3 are presented.

**Table 4:** Correlations between circRNA expression levels in HAPE patients and clinical variables.

	circ0058497	circ0083220	circ0081006
WBC	-0.051	0.477**	0.213*
LYMPHp	0.076	-0.202*	-0.166
GRANp	0.006	-0.059	0.023
HGB	-0.679**	-0.151	-0.412**
RBC	-0.601**	-0.150	-0.440**
HCT	-0.547**	-0.178	-0.361**
MCV	-0.280**	-0.179	-0.025
MCH	-0.278**	-0.053	0.026
MCHC	-0.034	0.147	0.073
PLT	0.104	0.226*	0.191
MPV	-0.005	-0.131	0.003
PCT	0.116	0.190	0.175
PLCR	0.008	-0.113	0.006
Cl	-0.015	-0.04	0.184
tCa	-0.155	0.026	-0.137
ALT	-0.19	-0.049	-0.111
AST	-0.239*	-0.269**	-0.251*
AST/ALT	0.069	-0.15	-0.079
TP	0.087	-0.014	-0.022
ALB	0.064	-0.033	-0.019
GLO	0.093	0.021	-0.019
A/G	-0.032	-0.045	0.028
TBIL	-0.275**	-0.039	-0.186
DBIL	-0.266**	0.065	-0.203*
IBIL	-0.334**	-0.159	-0.124
LDH	-0.13	-0.058	-0.166
UREA	0.019	0.048	-0.16

*P <0.05, **P<0.01, ***P<0.001

WBC, white blood cell; LYMPHp, lymphocyte percentage; GRANp, granulocyte percentage; HGB, hemoglobin; RBC, red blood cells; HCT, hematocrit; MCV, mean corpuscular volume; MCH, mean corpuscular hemoglobin; MCHC, mean corpuscular hemoglobin concentration; PLT, platelet; MPV, mean platelet volume; PCT, procalcitonin; PLCR, platelet-larger cell ratio; tCa, total calcium; ALT, alanine aminotransferase; AST, aspartate aminotransferase; TP, total protein; ALB, albumin; GLO, Globulin; A/G, albumin/globulin ratio; TBIL, total bilirubin; DBIL, direct bilirubin; IBIL, Indirect bilirubin; LDH, lactate dehydrogenase; UREA, urea levels.

### 3.7 Identification of DEGs in VECs

**Figure 7:**
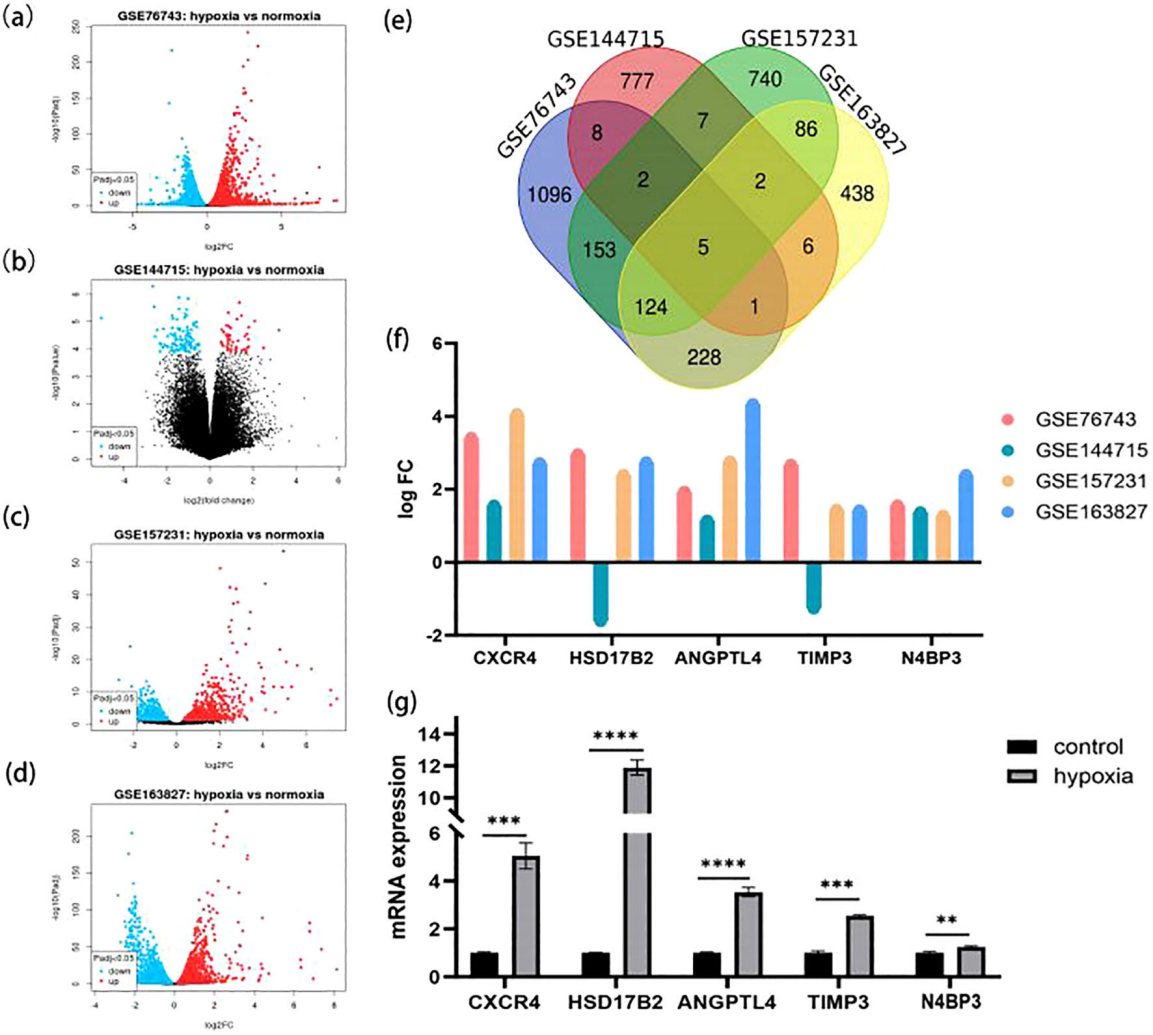
HAPE-related DEGs in vascular endothelial cells. **(A–D)** Volcano plots of DEGs within the four GEO datasets. **(A)** GSE76743; **(B)** GSE144715; **(C)** GSE157231; **(D)** GSE163827. **(E)** Venn diagram plot of overlapping DEG between the indicated datasets. **(F)** The intensity of the differential expression of the overlapped DEGs under normoxia and hypoxia in four datasets. **(G)** Bar graph showing the quantitative analysis of the rt-qPCR results. * P <0.05, ** P<0.01, ***P<0.001, ****P<0.0001.

Dysfunction of VECs is a driving force in the initiation and development of HAPE with exposure to hypoxia (20). We identified potential CAVD-related DEGs in VECs across datasets GSE76743, GSE144715, GSE157231, and GSE163827. Data were normalized and are presented in **Supplementary Figure S4**. DEGs have been identified and are displayed in **Figures 7A–D**. Following the extraction of the intersection of the four differential gene sets, five genes (CXCR4, HSD17B2, ANGPTL4, TIMP3, N4BP3) were identified (**Figures 7E, F**). Moreover, of these five differentially expressed genes, three (CXCR4, HSD17B2, N4BP3) showed differential expression between the HAPE group and the control group. Finally, we verified five genes that exhibited differential expression in HUVECs under normoxia and hypoxia via rt-qPCR analysis (**Figure 7G**).

## 4 Discussion

High altitude pulmonary edema (HAPE) is a complex disease that specifically arises in the hypobaric hypoxia conditions found at high altitudes. Recent advances in high-throughput sequencing technologies have allowed for extensive investigations into the pathogenesis of HAPE, enhancing our understanding of its molecular and genetic underpinnings. Lining Si et al. were the pioneers in identifying a correlation between polymorphisms in the CYP4F2 gene and the risk of HAPE in the Chinese Han population (21). Chen et al. advanced this research by employing whole-exome sequencing (WES) to pinpoint genetic variations linked to HAPE in 50 unrelated patients (22). Another significant contribution came from a study that sequenced the entire mitochondrial genome of individuals who are either resistant or susceptible to HAPE within the Han Chinese population, uncovering two non-coding variants in the mitochondrial genome that vary in frequency between the groups (23). Furthermore, Kavita Sharma et al. discovered 30 SNPs in two crucial hypoxia-related genes (EGLN1 and HIF1AN), which may have functional implications for Indians either susceptible to or protected from HAPE (24). Despite significant advances in HAPE research, the etiology and pathogenesis of this condition are still not fully understood.

Whole transcriptome sequencing has been instrumental in uncovering genetic variations in complex and rare cell populations and in elucidating regulatory gene relationships. This enhances our understanding of the molecular mechanisms that underpin disease onset and progression. Our study utilized RNA-seq data to identify regulatory mRNAs, miRNAs, lncRNAs, circRNAs, alternative splicing (AS) events, fusion genes, and novel transcripts in the blood samples from HAPE patients and controls. We also conducted an analysis of immune cell infiltration and investigated key genes linked to endothelial cell dysfunction in HAPE. Importantly, we selected three key circRNAs—hsa_circ_0058497, hsa_circ_0081006, and hsa_circ_0083220—for RT-qPCR validation on a larger sample set. This enabled us to ascertain their relationships with clinical parameters and assess their potential as diagnostic biomarkers.

While the role of miRNAs in the development and progression of high-altitude sickness has been extensively explored, our understanding of their involvement in HAPE is still limited. Notably, studies identified a correlation between HAPE and altered levels of circulating miRNAs, suggesting a potential biomarker or mechanistic pathway in the disease process. For instance, Alam P et al. discovered significant overexpression of miR-124-3p in HAPE patients, which likely played a crucial role in the pathophysiology of HAPE (1). Under hypoxic conditions, miR-424/322 can regulate the HIF-1α-VEGF axis and prevent pulmonary vascular leakage (25). In our study, hsa-miR-143-3p, hsa-miR-708-5p, hsa-miR-1306-5p, hsa-miR-760, hsa-miR-150-5p, and hsa-miR-342-5p were identified within the ceRNA networks. A study demonstrated that inhibiting miR-143-3p expression can promote growth and metastasis of adenocarcinoma cells in the lung (26). Another study showed that miR-143-3p is associated with inflammation and pain reactions. This miRNA affects pulmonary inflammatory factors and cell apoptosis in mice with mycoplasma pneumonia by regulating the TLR4/MyD88/NF-κB signaling pathway (27). Yang et al. found that miR-760 mediates hypoxia-induced proliferation and apoptosis in human pulmonary arterial smooth muscle cells by targeting TLR4 (28). A study confirms that miR-150-5p, associated with IL-6, IL-15, IL-17A, and IL-1β, is highly correlated with monocyte chemotactic protein-1 (MCP-1) and can be used to identify early infections (29). Furthermore, hsa-miR-150-5p is significantly linked to drug resistance and survival in non-small cell lung cancer (NSCLC) via the ceRNA mechanism (30). MiR-342-5p, targeting the SARS-CoV-2 gene (ORF1ab) and several host genes, plays a key role in SARS-CoV-2 infection (31). The aforementioned miRNAs are closely related to lung diseases, underscoring their potential undeniable role in HAPE. Research on the roles of miR-708-5p, miR-1306-5p, and miR-342-5p in lung diseases has not yet been reported.

However, to the best of our knowledge, no HAPE-associated circRNAs and lncRNAs have been identified in human samples to date. Here, we present the first differential expression analysis of circRNA and lncRNAs in blood samples from patients with HAPE. Interestingly, it is worth noting that from large sample validation results, hsa_circ_0058497, hsa_circ_0081006, and hsa_circ_0083220 not only demonstrated significant expression differences between the two groups, but also exhibited high diagnostic value and clinical relevance. These circRNAs were upregulated according to microarray and rt-qPCR analysis. By analyzing the area under the ROC curve of blood samples, we determined that hsa_circ_0081006 had the highest diagnostic value for HAPE among all tested circRNAs and showed a significant positive correlation with WBC (r = 0.477, P < 0.01). Therefore, our findings suggest that hsa_circRNA_0081006 may be involved in regulating white blood cell proliferation and immune system function in HAPE. In addition, hsa_circ_0058497 and hsa_circ_0083220 showed diagnostic value and were negatively correlated with red blood cells, hemoglobin, and hematocrit. In particular, hsa_circ_0058497 correlated with red blood cells and hemoglobin, both above 0.6. Hemoglobin, a protein-based component of red blood cells, is primarily responsible for carrying oxygen from the lungs to the respiratory tissues, transporting it through the bloodstream, and maintaining oxygen levels in biological tissues (32). Hematocrit, which reflects blood health, is the percentage of red blood cells in the blood (33). In plateau environments where oxygen is scarce, the body increases the oxygen-carrying capacity of the blood by raising the number of red blood cells and hemoglobin to maintain an adequate oxygen supply. This adaptive response results in increased blood viscosity, potentially leading to microcirculatory disturbances, thrombosis, and extensive organ damage (34). Thus, our results suggest that hsa_circ_0058497 and hsa_circ_0083220 may relate to functions such as erythropoiesis and oxygen transport in HAPE patients.

This study demonstrated that the HIF-1 and VEGF signaling pathways, crucial for angiogenesis, were predominantly enriched in pathways associated with HAPE. Multiple lines of evidence suggest that the HIF pathway is primarily responsible for high-altitude adaptation. HIF-1α plays a central role in the HIF-1 signaling pathway. HIF1β and HIF1α combine to form the heterodimeric transcription factor known as HIF1, the primary mediator of cellular responses to hypoxia (35). The genes of the hypoxia-inducible factor pathway have been linked to adaptations in species living at high altitudes, including both humans and non-humans (36). EGLN1 and EPAS1, crucial HIF regulators, are frequently identified as targets of selection in Tibetan adaptation to high altitudes (37). Additionally, HIF1α is known to induce VEGF expression. The VEGF pathway, downstream of HIF-1, significantly contributes to high-altitude adaptations. Under hypoxic conditions, HIF activity increases and can bind to the VEGF gene promoter, promoting its transcription and translation, thereby increasing VEGF levels (38). Furthermore, it has been demonstrated that VEGF can impact HIF activity. Specifically, research indicates that VEGF can enhance HIF stability through activation of the PI3K/Akt signaling pathway. The results imply that these pathways may play an important role in the pathogenesis of HAPE.

Alternative splicing (AS) events, fusion genes, and novel transcripts represent important forms of gene expression in organisms. They play roles in a wide array of physiological and pathological processes. Currently, research on the relationship between these phenomena and high-altitude diseases is limited. However, some studies suggest that alternative splicing and novel transcripts may contribute to the occurrence and development of high-altitude diseases. For instance, a study of peregrine falcons revealed that populations on the Qinghai-Tibet Plateau possess more moderately to highly expressed hemoglobin transcripts, potentially advantageous for better adaptation to the hypoxic environment at high altitudes (39). However, further validation of these findings remains necessary, and additional research is required to elucidate the specific association between these phenomena and high-altitude diseases. This study offers insights into the potential contributions these phenomena may make to HAPE in the future.

Recent research indicates that inflammation may play a crucial role in the development of HAPE. The inflammatory response in HAPE patients was significantly enhanced compared to controls, evidenced by a marked increase in peripheral leukocyte counts and serum levels of IL-1β, TNF-α, IL-6, and IL-8 (40). During inflammation, immune cells release chemicals such as cytokines and chemokines, which attract additional immune cells to the site, forming an immune cell infiltration. Our results from immune cell infiltration analysis suggest that HAPE is associated with multiple immune cell types. T follicular helper (Tfh) cells are a subset of CD4-positive helper T cells that play a crucial role in the humoral immune response. Their primary function is to induce germinal center B cell differentiation into antibody-secreting plasma cells and memory B cells (41). Thus, Tfh cells enhance the humoral immune response. Macrophages are critical components of the immune system, capable of phagocytosing and killing pathogens, as well as secreting various cytokines to regulate immune responses. Based on their function and activation, macrophages can be divided into two subtypes: classically activated M1 macrophages and alternatively activated M2 macrophages. M1 macrophages are mainly responsible for recruiting Th1 cells, providing resistance to pathogens, and controlling tumors through natural and adaptive immune responses (42). B cells are crucial components of the immune system, responsible for producing antibodies to combat pathogens. B cells can be classified as either naive or memory B cells, depending on their maturity and antigen exposure. Naive B cells and memory B cells both play distinct roles in the immune response. Naive B cells secrete few cytokines, but activated B cells can produce a range of pro- and anti-inflammatory cytokines that affect immune function in various ways (43). Memory B cells are characterized by their ability to retain information about the original antigen over a long period. Upon re-encountering the same antigen, they can rapidly produce a significant quantity of antibodies, thereby providing long-lasting immune protection (44). Therefore, B cells, which fight pathogens, regulate immune responses, and provide long-term immune protection, are critical to the onset and progression of HAPE. Overall, due to their role in regulating inflammatory and immune responses, immune cell infiltration may play a critical role in the development of HAPE. However, further research is needed to better understand the processes involved.

Furthermore, hypoxia-induced pulmonary endothelial cell dysfunction has been linked to lung diseases such as HAPE and pulmonary arterial hypertension. At high altitudes, the lack of oxygen can damage pulmonary endothelial cells, leading to structural damage of the blood vessel wall. This can increase vascular permeability, allowing water and electrolytes from the blood to enter the alveoli, culminating in HAPE. This study investigates the key genes (CXCR4, HSD17B2, ANGPTL4, TIMP3, N4BP3) responsible for endothelial cell dysfunction under hypobaric and hypoxic conditions. It has been reported that CXCR4 expression is downregulated in the evolutionarily selected Tibetan prolyl hydroxylase-2 variant, which degrades HIF α more effectively, thereby protecting highlanders from the adverse effects of increased hemoglobin concentration due to hypoxia (45). HSD17B2 regulates the levels of active sex steroids in the lungs and prevents their overabundance (46). Plasma biomarkers were measured for mountaineers suffering from high-altitude diseases and healthy adapted mountaineers. The results showed that ANGPTL4 was associated with AMS and HAPE, but not with severity (47). The minor allele C of rs130293 (C/T) in the TIMP3 gene has been associated with resistance to HAPE, while the major allele T has been associated with susceptibility to HAPE, according to a genome-wide association study (48).

Our research raises several questions. Is it possible to follow the progression of HAPE using whole blood transcriptional characteristics? Our data indicate that there is one cluster of genes associated with abnormally low pulmonary function and another cluster associated with high-altitude hypoxia. Their usefulness in monitoring disease progression and severity must be validated in longitudinal studies. We also question whether peripheral blood transcriptional signals could be used to track response to therapy. This will be the focus of our future research. Finally, we question whether our study shows heterogeneity in the characteristics of unknown genes that impact the onset and development of HAPE. Our research was the first to identify circRNAs, lncRNAs, AS events, fusion genes, and novel transcripts.

This study has several limitations that warrant consideration: 1) Sample Size: While our study validated findings with two sets of 50 samples each—a reasonable size in comparison to many biomedical studies—larger sample sizes could yield more stable and reliable results, especially in complex diseases and multivariate analyses. Small sample sizes are a common issue in high-altitude medicine research. We are in the process of collecting blood samples from a larger population for further validation. 2) ceRNA Network Validation: The ceRNA networks in this study were constructed based on computational predictions and statistical correlations. These networks require further validation through experimental methods to confirm the interactions and functional impacts suggested by our computational analyses. 3) Translation to Clinical Practice: There is a significant gap between basic research and clinical application. Although we identified certain RNA molecules and gene expression patterns associated with HAPE, additional validation is necessary to determine whether these can be effectively transformed into clinical diagnostic markers or therapeutic targets. This step is crucial for translating our findings into practical clinical applications that can improve patient outcomes.

## 5 Conclusion

For a deep mechanistic analysis of the pharmacological effects of HAPE, whole transcriptome sequencing and other techniques to determine crosstalk between mRNAs, miRNAs, lncRNAs, and circRNAs were effectively used in this study. Since we found specific circRNAs in the blood that correlate with gene expression patterns in high-altitude sickness and lung function abnormalities, these data support future studies on blood biomarkers for disease progression and phenotyping in HAPE, potentially aiding clinical trials. By comparing transcriptome data from disease and normal samples, this study will elucidate the mechanisms of gene expression regulation, discover new transcripts and genes, expand our understanding of the transcriptional features of HAPE, identify potential disease markers, reveal disease-related pathways and biological processes, and provide insights for future research.

## Data Availability

The datasets presented in this study can be found in online repositories. The names of the repository/repositories and accession number(s) can be found below: GSE260910 (GEO).

## Supplementary material

Supplementary Figure 1
**(A–D)** The Heatmaps of the DEmRNA, DEmiRNA, DEcircRNA, DElncRNA. **(A)** DEmRNA; **(B)** DEmiRNA; **(C)** DEcircRNA; **(D)** DElncmRNA.

Supplementary Figure 2 Functional enrichment analysis of GO and KEGG in HAPE. **(A–D)** Bar charts of differential gene GO enrichment. The longer the P-value line in the figure, the larger the P-value, and the less significant it is. Purple: MF (molecular function); Red: CC (cellular component); Yellow: BP (biological process); **(A)** DEmRNA; **(B)** miRNA target genes; **(C)** the source genes for circRNAs; **(D)** lncRNA target genes; **(E–H)** Dot plots of KEGG pathway enrichment analyses.**(E)** DEmRNA; **(F)** miRNA target genes; **(G)** the source genes for circRNAs; **(H)** lncRNA target genes.

Supplementary Figure 3 Data preprocessing and normalization. **(A, B)** The principal component analysis (PCA) plots for all batches. **(A)** before removing effects; **(B)** after removing effects; **(C, D)** box plots showing data normalized. **(C)** before data normalized; **(D)** after data normalized.

Supplementary Figure 4 Data normalization in GEO datasets. **(A)** GSE76743; **(B)** GSE144715; **(C)** GSE157231; **(D)** GSE163827.
