# Supplementary figures and images for: Whole transcriptome landscape in HAPE under the stress of environment at high altitudes: new insights into the mechanisms of hypobaric hypoxia tolerance
Source: Front Immunol. 2024 Sep 12;15:1444666. doi: 10.3389/fimmu.2024.1444666 (PMC11424462; doi:10.3389/fimmu.2024.1444666)

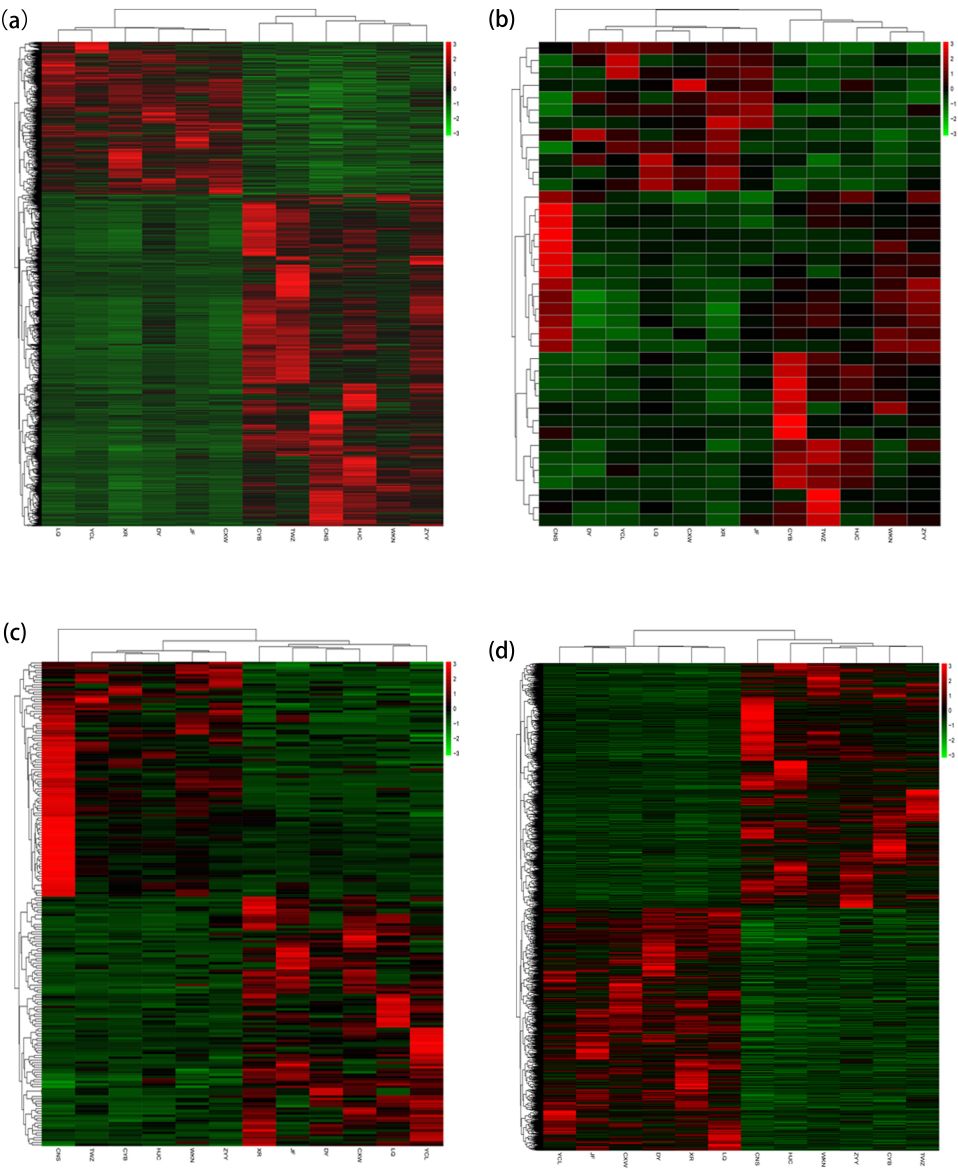

Supplement: Supplementary Figure 1 — (A–D) The Heatmaps of the DEmRNA, DEmiRNA, DEcircRNA, DElncRNA. (A) DEmRNA; (B) DEmiRNA; (C) DEcircRNA; (D) DElncmRNA. [file Image1.jpeg]

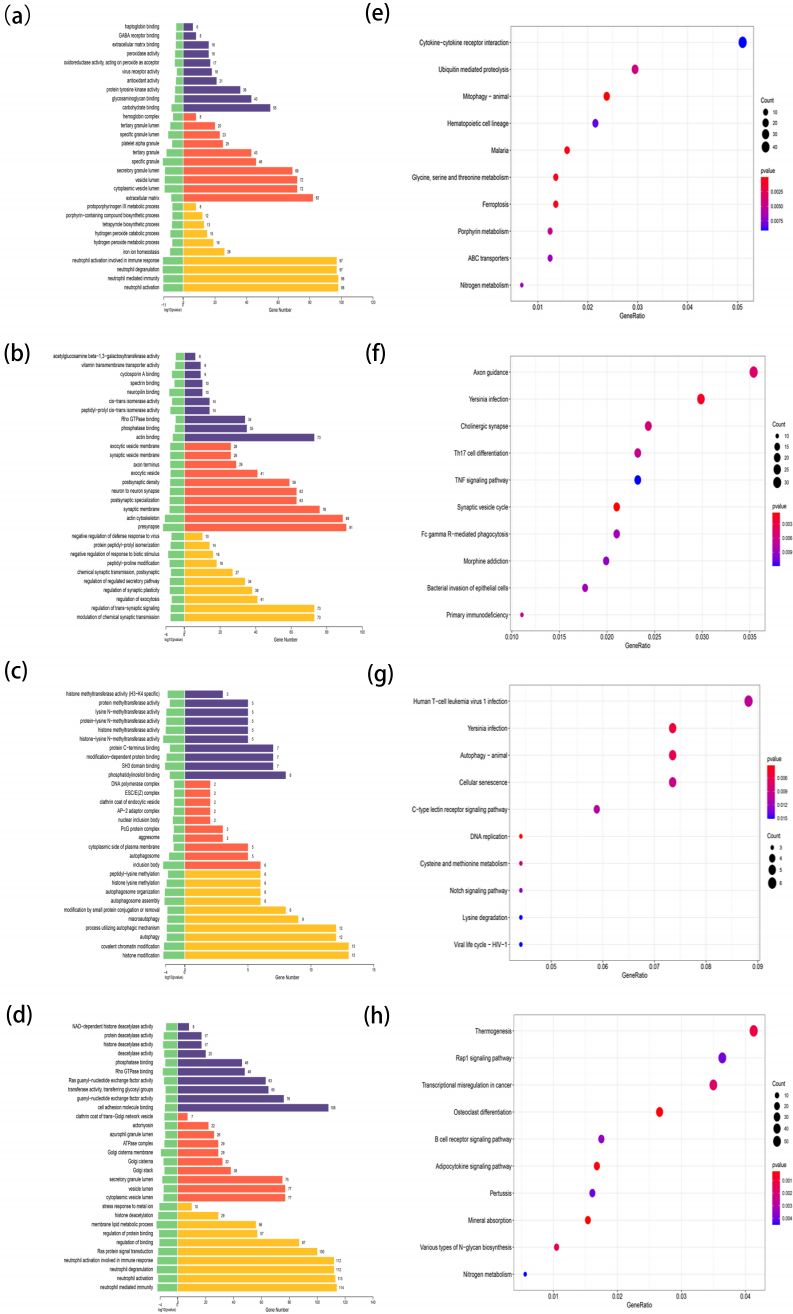

Supplement: Supplementary Figure 2 — Functional enrichment analysis of GO and KEGG in HAPE. (A–D) Bar charts of differential gene GO enrichment. The longer the P-value line in the figure, the larger the P-value, and the less significant it is. Purple: MF (molecular function); Red: CC (cellular component); Yellow: BP (biological process); (A) DEmRNA; (B) miRNA target genes; (C) the source genes for circRNAs; (D) lncRNA target genes; (E–H) Dot plots of KEGG pathway enrichment analyses.(E) DEmRNA; (F) miRNA target genes; (G) the source genes for circRNAs; (H) lncRNA target genes. [file Image2.jpeg]

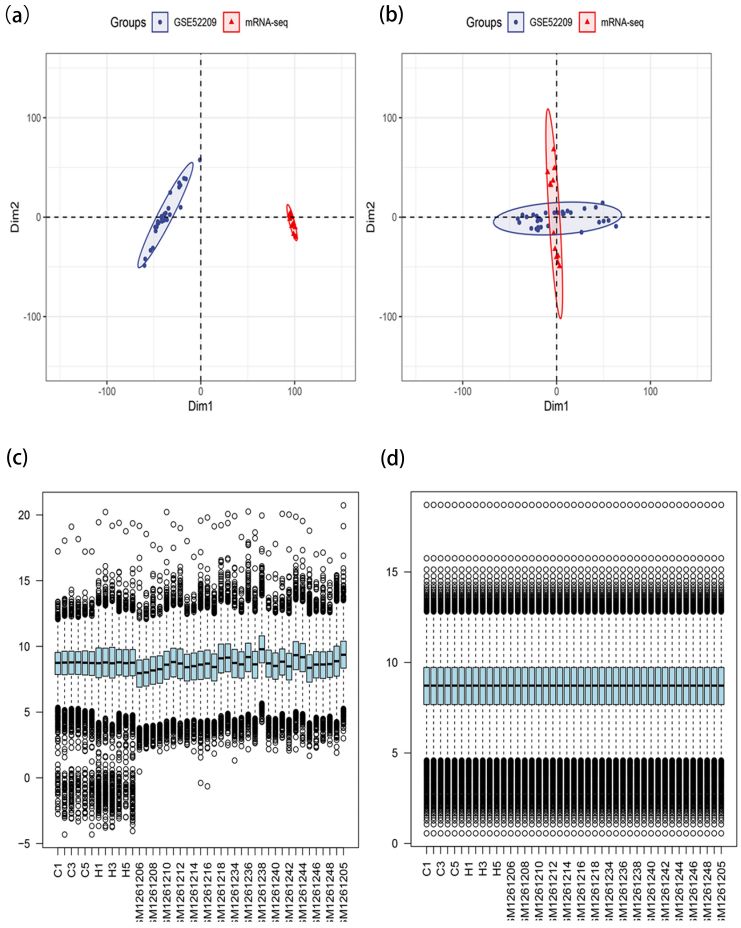

Supplement: Supplementary Figure 3 — Data preprocessing and normalization. (A, B) The principal component analysis (PCA) plots for all batches. (A) before removing effects; (B) after removing effects; (C, D) box plots showing data normalized. (C) before data normalized; (D) after data normalized. [file Image3.jpeg]

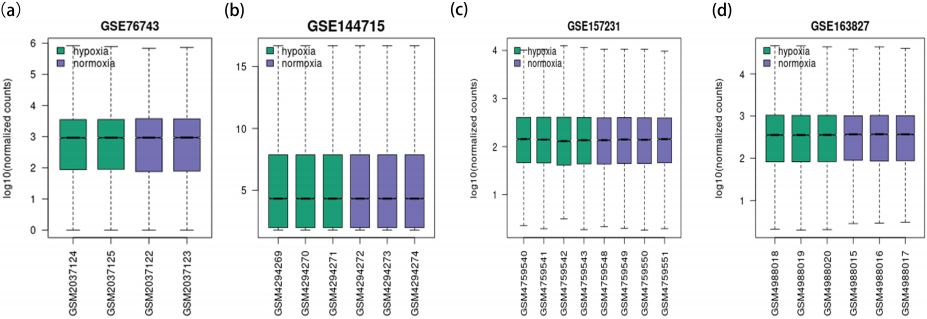

Supplement: Supplementary Figure 4 — Data normalization in GEO datasets. (A) GSE76743; (B) GSE144715; (C) GSE157231; (D) GSE163827. [file Image4.jpeg]
